# Oxidative stress as a shared mechanisms for different prenatal stressors: long-term effects on adolescent male and female mouse offspring

**DOI:** 10.1192/j.eurpsy.2023.294

**Published:** 2023-07-19

**Authors:** C. Musillo, A. Berry, K. C. Creutzberg, B. Collacchi, M. Samà, L. Giona, M. A. Riva, F. Cirulli

**Affiliations:** 1 Istituto Superiore di Sanità, ROMA; 2 Università degli Studi di Milano, Milan, Italy

## Abstract

**Introduction:**

Stressful experiences *in utero* can produce physiological changes which become embedded biological traces affecting fetal brain development and ultimately leading to increased vulnerability for psychiatric disorders.

**Objectives:**

We hypothesized that stressors as diverse as maternal obesity and maternal psychophysical stress might disrupt fetal programming resulting in long-lasting effects on offspring brain development by acting through shared oxidative stress (OS)-mediated mechanisms.

**Methods:**

We compared a mouse model (C57Bl/6N) of maternal high-fat diet (HFD) consumption (13 weeks, until delivery) to prenatal restraint stress (PNS) repeatedly administered during the last week of pregnancy. To counteract the negative effects of both stressors, the antioxidant N-acetyl-cysteine (NAC, 1 g/kg) was administered to female breeders for 8 weeks until delivery. Emotionality was assessed in adolescent male and female offspring through the elevated-plus-maze (EPM). Moreover, hippocampal gene expression levels of Brain-Derived-Neurotrophic-Factor (*Bdnf* ), Nuclear factor erythroid 2–related factor 2 (*Nrf-2*) and Kelch-like ECH-associated protein 1 (*Keap-1*) were measured, by qPCR, as markers of brain plasticity and antioxidant capacity.

**Results:**

Prenatal exposure to both HFD and PNS enhanced behavioral disinhibition, increasing time spent in the open arms of the EPM and decreasing the frequency of risk-assessment behaviors, especially in female offspring. Moreover, both prenatal stressors led to decreased *Bdnf* (in females) and *Nrf-2* levels, and disrupted *Keap-1* levels. Prenatal NAC was able to counteract these effects on the brain.

**Conclusions:**

Our data support the hypothesis of a “funnel effect” model explaining how different prenatal stressors result in long-term negative effects on the adolescent offspring, increasing risk assessment behaviors and affecting brain plasticity and antioxidant defenses. The beneficial preventive effects of NAC suggest that OS may be a common mechanism, playing a pivotal role in fetal programming of mental disorders. *ERANET-NEURON-JTC-2018-Mental Disorders-“EMBED” and Bando Ricerca Indipendente ISS 2021-2023; MOMINFLAM. Unique signatures underlying placental-fetal brain crosstalk in maternal obesity to F Cirulli.*

**Disclosure of Interest:**

None Declared

